# An application of BWM for risk control in reverse logistics of medical waste

**DOI:** 10.3389/fpubh.2024.1331679

**Published:** 2024-01-26

**Authors:** Xiaozhu Wang, Long Liu, Lingyu Wang, Wenjun Cao, Di Guo

**Affiliations:** ^1^School of Healthcare Technology, Dalian Neusoft University of Information, Dalian, China; ^2^Advanced Institute for Medical Sciences, Dalian Medical University, Dalian, China; ^3^Department of Thoracic Surgery, Zhongshan Hospital Affiliated to Dalian University, Dalian, China; ^4^School of Information and Business Management, Dalian Neusoft University of Information, Dalian, China

**Keywords:** medical waste logistics, public health emergencies, risk identification, risk assessment, best-worst method

## Abstract

The pollution posed by medical waste complicate the procedures of medical waste logistics (MWL), and the increasingly frequent occurrence of public health emergencies has magnified the risks posed by it. In this study, the authors established an index of the factors influencing the risks posed by MWL along five dimensions: the logistics business, emergency capacity, equipment, personnel, and management. The best-worst case method was used to identify the critical risk-related factors and rank them by importance. Following this, we assessed the risk posed by MWL in four major cities in China as an example and propose the corresponding measures of risk control. The results showed that the linking of business processes was the most important factor influencing the risk posed by MWL. The other critical risk-related factors included the location of the storage site, the capacity for emergency transportation, measures to manage emergencies, and the safety of packaging. Of the cities considered, Beijing was found to be a high-risk city, and its MWL needed to be improved as soon as possible in light of the relevant critical risks. Shanghai, Guangzhou, and Shenzhen were evaluated as general-risk cities, which meant that the risks of MWL were not a priority in these areas, and the other goals of urban development should be comprehensively considered during the long-term planning for MWL in these municipalities.

## 1 Introduction

Medical waste refers to waste containing infectious materials that is generated by institutions engaged in medical services, such as medical treatment, prevention, and healthcare (1). Past studies have shown that medical waste contains infectious pathogens, chemicals, and even radioactive substances. Medical waste contains dozens, and even hundreds, of times more germs that ordinary household garbage that can readily harm human health. For example, the WHO reported that abandoned needles cause about 1.6 million people to be infected with hepatitis B, 0.31 million people to be infected with hepatitis C and 0.03 million people to be infected with AIDS virus every year. Moreover, the packaging and containers used for medical waste contain a large amount of plastic, glass, and metal shards that are highly resistant to degradation. The long-time exposure of these materials to the environment has negative effects on the air, water, and soil. Furthermore, there are many kinds of medical waste, the methods of disposal of which are significantly different and complicated such that they require skilled professionals (2). The safe disposal of medical waste has attracted attention in research since the 1950s. The relevant theoretical work has helped refine the definition of medical waste, technologies for its disposal, and specifications of the disposal process (3). Many countries have also implemented special regulations on medical waste management and established disposal centers to ensure that medical waste is appropriately separated from domestic waste. The frequency, duration, and impact of global public health emergencies have increased in the past two decades (4). Such emergencies can cause a sharp increase in the medical waste generated in a short time, where this poses a daunting challenge to urban public health.

**Figure 1:**
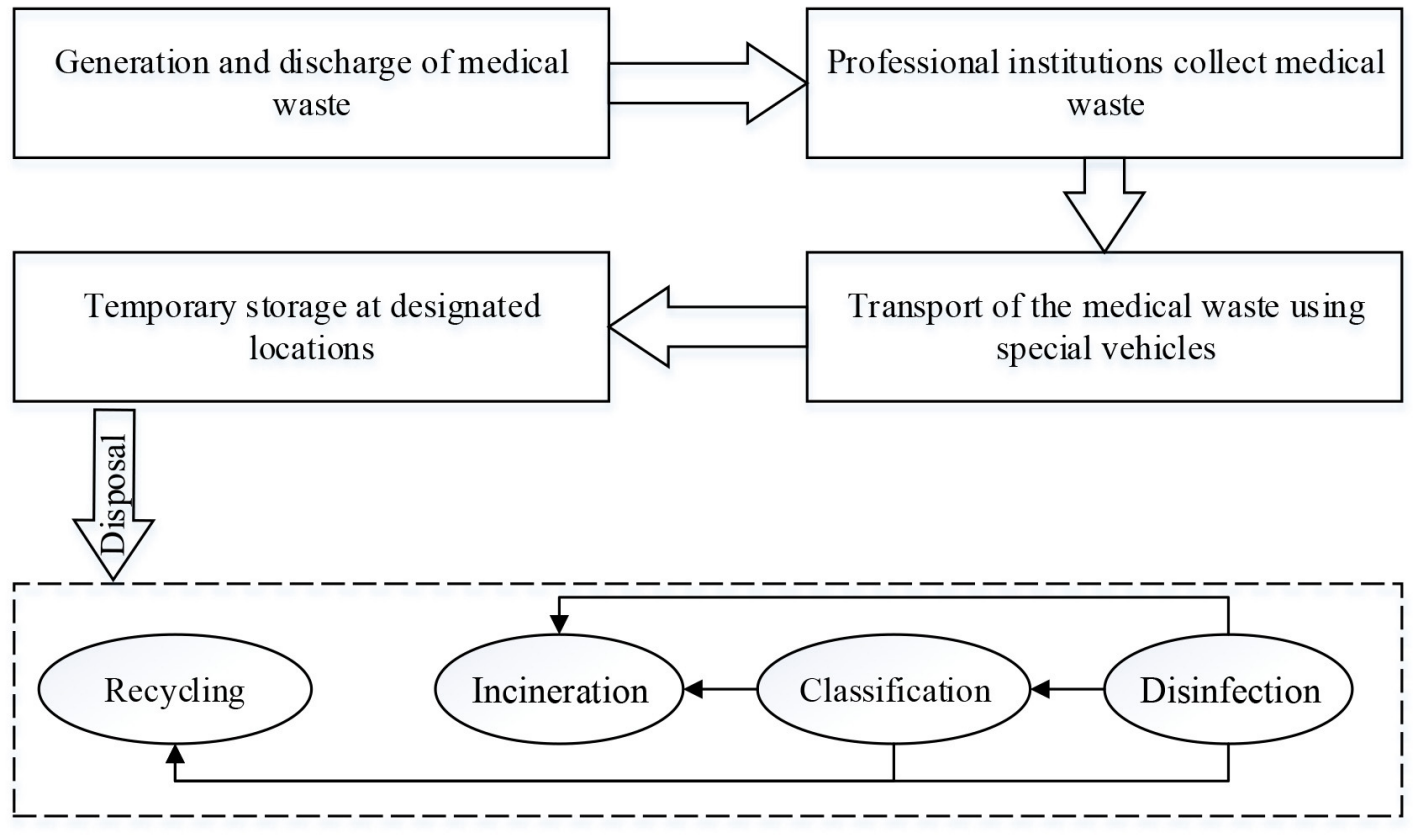
The process of medical waste logistics.

The process of disposal of medical waste is shown in Figure 1. First, the medical waste is discharged, packaged and placed in temporary storage sites. Second, the waste is transferred to special disposal centers in designated vehicles. Finally, it is subjected to high temperature incineration, steam disinfection, and chemical disinfection for disposal. The process of disposal of medical waste must be completed within 12 h. This process is thus complex, and needs to be quickly carried out. It is also likely that a large amount of newly generated medical waste cannot be disposed of in time when public health emergencies occur. Figure 1 shows the medical waste logistics (MWL) involves many participants, and accidents or disruptions in any one link of MWL can cause the entire system to collapse (5). For example, inappropriate packaging can cause contamination of vehicles, leading to the spread of pollution. An obstructed logistics network can lead to excessive inventory of medical waste. Insufficient logistics equipment will be unable to cope with the sudden increase in medical waste in public health emergencies. Therefore, managing the risk posed by MWL in case of public health emergencies is an important issue that requires further examination.

Researchers have investigated the disposal of medical waste generated in case of public health emergencies. Given the low capacity for the disposal of such waste, Xueyun studied the construction of temporary facilities and the requisitioning of domestic waste incineration centers (6). Joneghani et al. (7) analyzed the risk posed by medical waste to public safety, the urgency of its disposal, and the cost of its disinfection, and used this information to optimize the location of sites of the waste disposal network to increase its efficiency (7). Mohamed Faizal et al. (8) examined optimizing the path of the network to reduce congestion in the transportation network used to dispose of medical waste generated in case of public health emergencies. Researchers have also studied the responses to and aftercare plans in light of the risks posed by medical waste generated during public health emergencies (5). The above shows that a considerable amount of research has been conducted on the classification, collection, transportation, storage, and disposal of medical waste in case of public health emergencies, but little work has been reported on managing the risk in this case based on a systematic perspective. Because of the public benefit provided by MWL, its coordination and implementation cannot be left to the mercy of the market mechanism. This article identifies and controls key risk factors in urban MWL from the perspective of government macroeconomic regulation based on the theory of integrated supply chain management.

This study seeks to answer the following questions: how do we ensure that only a limited amount of resources can be used to control the critical risks posed by MWL by identifying them? How do we evaluate the risk of MWL in areas in which public health emergencies have occurred based on a system of risk-related factors? How do we design effective measure of risk control based on these factors? Answering these questions can help urban health management departments to reasonably integrate their resources and improve MWL, and to design mechanisms of coordination for the participants of MWL. This can in turn improve the capacity of the city's response to public health emergencies, and protect the urban environment as well as public health. We assume here that the relevant decisions need to be made under limited resources, the relevance and importance of different types of risk-related factors need to be assessed, and the available information needs to be used to evaluate the alternatives at hand. This is a typical multi-criteria decision-making (MCDM) problem. We used the best-worst method (BWM) to identify the risk-related factors. We also evaluated the risk of MWL in four first-tier cities of China.

The remainder of this paper is organized as follows: Section 2 reviews the literature on identifying, assessing, and controlling risks in MWL, and Section 3 introduces the proposed method. Section 4 considers four cities in China as an example of how to establish a system of indicators of risks posed by MWL, measure the weights of the key risk-related factors, and evaluate the overall risk. Section 6 provides a discussion of the results and the conclusions of this study.

## 2 Literature review

For a literature review, this study implemented four steps processes as prescribed under the qualitative content analysis method: material collection, descriptive analysis, category selection and finally, material evaluation (9). Firstly, keywords such as “medical waste,” “medical waste logistics,” “risk factor of medical waste logistics,” “factor identification,” “factor evaluation” and “risk control” were used in the Scopus (www.scopus.com), and Web of Science (WoS) databases to collect materials. Secondly, the collected materials should be analyzed by year and journal to ensure the quality of the literature. Then, the collected materials should be classified into different research categories. Finally, all necessary information should be recorded, evaluated and organized into the literature review.

### 2.1 The importance of MWL

In recent years, frequent public health emergencies have led to an increase of medical waste, putting enormous pressure on the waste management system (10). Medical wastes cause infectious diseases (11). According to the statistics, approximately 15 % of medical waste contains infectious, toxic, or radioactive substances (12). Improper disposal of waste generated by healthcare facilities can have a direct impact on human health and the environment (13). WHO recommends that medical waste be collected, stored, and disposed of separately from domestic and industrial wastes by accounting for its risks and threats (14). Medical waste management is actually a reverse logistics management problem. When we examine MWL practices, it is clear that MWL problems have not been solved in many developing countries yet. improper MWL procedures can aggravate the problem, leading to more severe risks to the sustainability of the environment and public health (11), such as mutagenic and carcinogenic problems, respiratory injuries, complications of the central nervous system, and reproductive system damage (15).

Therefore, research on MWL is crucial. By designing a reverse logistics network for medical waste, potential risks, including virus transmission, can be reduced. By utilizing risk control measures, the city's ability to respond to public health emergencies can be effectively improved, protecting the urban environment and human health.

### 2.2 Risk factors' identification and evaluation of MWL

The logistical process of the removal of medical waste includes its collection, storage, transportation, and treatment. Because medical waste may be highly spatially infectious, latent, and viral, many risks are involved in its logistics. Many scholars have studied risk-related factors in the process of MWL. Makajic-Nikolic et al. (16) studied the risks associated with the probability of generation of medical waste during diagnosis and treatment as well as during its transportation (internal and external) (16). Tushar et al. (2) studied the risks posed by medical waste from four perspectives: operation, technology, government, and management (2). Tang et al. (17) used the characteristics of transportation of medical waste combined with past research to propose 16 risk-related factors, including poor safety awareness among employees, incorrect operation by them, improper disinfection of transportation tools, unsafe transportation tools, and unreasonable storage locations. However, they did not classify these risk-related factors. Niyongabo et al. (18) studied risk-related factors involved in the generation and storage of medical waste in 12 healthcare facilities in Bujumbura, Burundi, and concluded that the risk posed by the storage process was the highest (18). The application of advanced technologies to MWL has also gradually been attended to in research. Celik et al. (19) claimed that the failure to use such new technologies as big data, the blockchain, and novel and reliable software will introduce risks to the process of MWL (19). The above literature shows that not many studies have been devoted to investigating and classifying the risk-related factors of MWL, where this can help control and reduce risks.

Identifying and evaluating risk-related factors in MWL is the key to managing the risk, and is important for improving medical waste management. Many scholars have studied risk identification and evaluation (16, 17, 19), but few have specifically focused on MWL. And the identification and evaluation of risk is a typical MCDM problem. The technology for multi-standard analysis can help decision-makers evaluate and control risks (20). Karuppiah et al. (21) used MCDM to establish a Framework for improving supply chain sustainability (21). Many scholars have used MCDM to examine the problem of risk management in different fields. Koohathongsumrit et al. (22) integrated the fuzzy risk assessment model (FRAM) and the best–worst method (BWM) to select the best transportation route in a multi-modal supply chain (22). Aydin et al. (23) proposed a framework for risk assessment based on the failure mode and effect analysis (FMEA), the best–worst method (BWM), and image fuzzy multi-attribute boundary approximation area comparison (PF-MABAC) to prioritize the risks of fires and explosions in the oil and gas industry (23). Celik et al. (24) combined the BWM and MARCOS to identify and analyze risks to the safety of dams (24), Zheng et al. (25) used the G-DEMATEL-AHP to study the risk of urban flooding (25), and Ghorui et al. (26) used the HFS and TOPSIS to identify major risk-related factors involved in the transmission of COVID-19 (26). The MCDM method has also been widely used in research on medical waste management, particularly in studies on techniques of management, and methods and sites of disposal (27–29).

The above shows that a diversity of methods to identify and assess risks have been developed. MCDM is a classic method of risk identification and assessment, and the BWM has been applied to manufacturing, transportation, and supply chain management (30–33). It is frequently used to derive the weights of the decision-related criteria in MCDM problems. Therefore, BWM is suitable for use in research on the risk management of MWL.

### 2.3 Research on risk control of MWL

With increasing public awareness of the impact of green development, disposing of medical waste in a safe and environmentally friendly manner has become a popular subject of research in recent years (19). Medical waste mainly affects healthcare workers, patients and their companions, cleaning personnel, and personnel engaged in the transportation and disposal of medical waste. It can also affect the environment, soil, water, and air. Therefore, managing and controlling the risks posed by it have attracted worldwide academic attention. Some researchers have designed logistics networks that can select the location of storage and treatment sites, and have planned and designed transportation routes to reduce the risks posed by MWL (34–37). The facilities and equipment used for MWL are crucial. Many studies have pointed out that the safety of the packaging and transportation of medical waste are key factors influencing its leakage (17). The operator is also important in MWL. Any non-compliance incurs unexpected risks. Some scholars have studied risk management in MWL from the perspective of the operators. For example, Makajic-Nikolic et al. (16) examined the risks caused by operators and their probability of occurrence, and concluded that better training of the personnel engaged in MWL and improving their awareness of safety can reduce risks (16). Moreover, the role of managers in MWL cannot be ignored (2). Some scholars have also studied the risk management of MWL in the context of public health emergencies in recent years. Chen et al. (38) used Wuhan, China as an example to study the management and control of the risks posed by the generation, transportation, and treatment of medical waste in emergencies, and their results showed that the emergency capacity needs to be improved (38). Liu et al. (39) claimed that the explosive growth in the volume of medical waste leads to risks due to limitations on the disposal facilities of hospitals when medical institutions respond to public health emergencies. It is thus crucial to design a reverse supply chain network of medical waste (39). Celik et al. (19) aimed to determine the hospital in Erzurum that carries out medical waste management the most effectively and efficiently by IFMCDM methods. Shanshan (40) summarized and analyzed methods of managing the risk of medical waste in China to reduce the risks posed by logistics (40). Nimita Jebaranjitham et al. (41) studied the impact of governmental policies on managing the risk of MWL, and concluded that Asian countries need to allocate more funds for the treatment of medical waste (41).

To sum-up, the process of risk control of MWL mainly involves the design of the reverse logistics network, management of the logistics operator and the equipment, formulation and supervision of a management system, and risk management in case of public health emergencies. Management based on the above perspectives can help control the risks posed by MWL.

### 2.4 Prototype decision-making structure

We chose and integrated the risk-related factors involved in MWL based on the above literature review and classified them into different aspects. We then eliminated factors that had the same meaning. We thus developed a prototype of a decision-making structure consisting of five aspects: (i) logistics, (ii) reserve for emergency capability, (iii) equipment, (iv) personnel-related risk, and (v) management-related risk. A detailed description of each category is provided in Table 1.

**Table 1:** The initial set of criteria for MWL.

**Aspect**	**Criteria**	**References**
Logistics	Transportation route selection	(17, 42, 43)
	Location of storage site	(5, 17, 44, 45)
	Controllability of in-transit time	(19, 43)
	Entity collaboration	(16)
	Information transmission channel	(2, 19, 46)
	Conversion and connection of business links	(16)
Emergency capability reserve	Transit capacity reserve	(38)
	Processing capacity reserve	(38, 39)
	Collection and classification capacity reserve	(38, 39)
	Storage capacity reserve	(2, 38, 39)
Equipment	Packaging safety	(16, 47)
	Transport safety	(16, 44, 48–50)
	Storage site security	(5, 17, 40, 44, 46)
	Safety of processing equipment	(16, 40)
	Progressiveness tools	(51, 52)
Personnel risk	Mastery of professional skills	(44, 49, 50, 53)
	Personnel protection awareness	(16, 43, 50)
	Popularity of laws and regulations	(40, 52)
	Professional ethics	(16, 40)
Management risk	Special management laws and regulations	(19, 47, 53)
	Regulatory mechanism	(43, 47, 49, 53–55)
	Emergency management measures	(17, 49, 55)
	Process standardization	(19, 47, 53, 55)

## 3 Methodology

**Figure 2:**
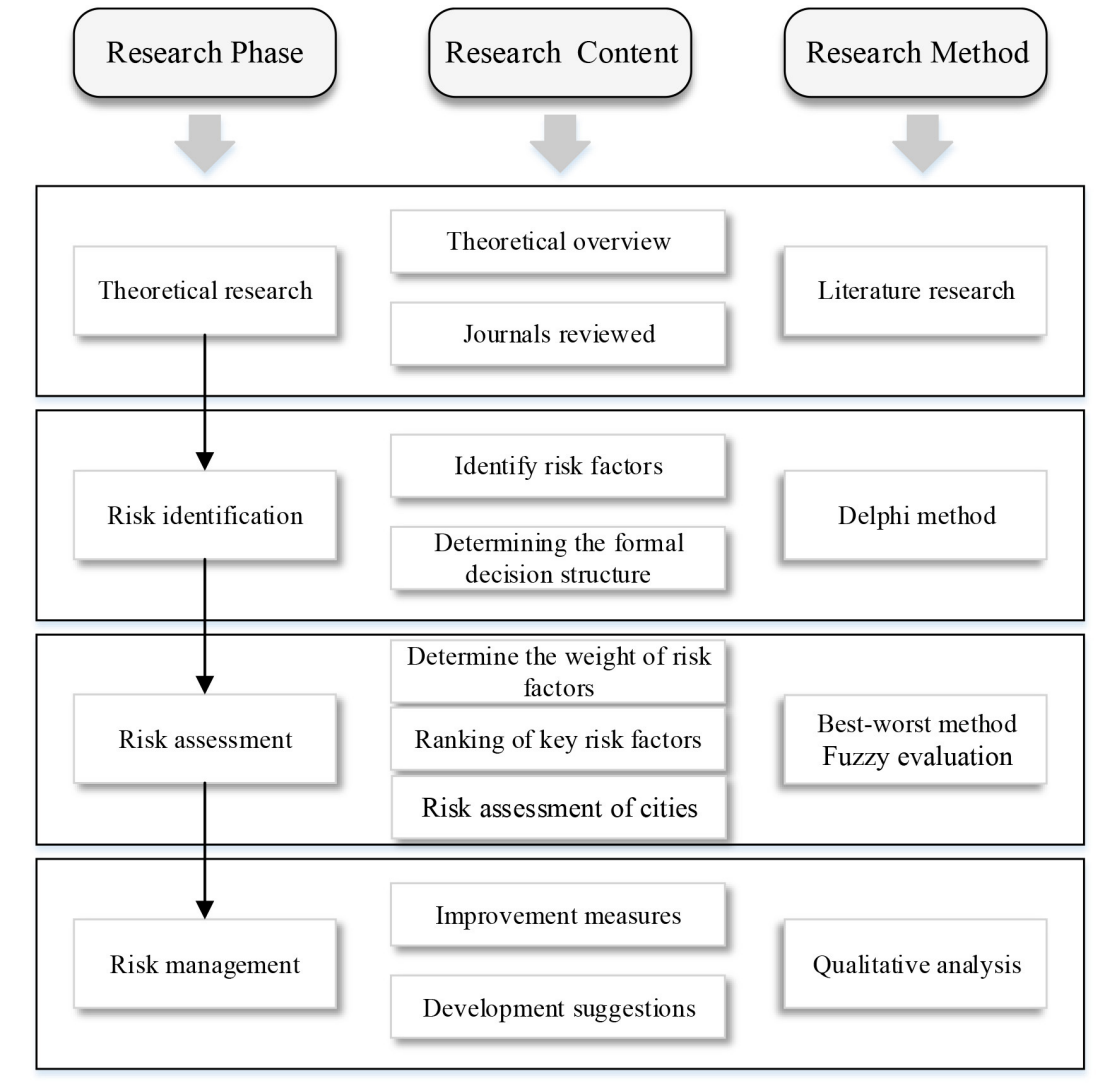
The description of the methodological framework.

Based on theoretical research, expand according to the logic of risk identification, risk assessment, and risk management. In Risk identification, the Delphi method is used to obtain the formal decision structure. In Risk assessment, BWM is used to calculate the weights of each criterion, and key factors are determined by ranking them based on the weights. Then, a Fuzzy evaluation was used to evaluate the performance of the four cities and obtain the final results. In risk management, based on the research results, propose management suggestions and directions. The description of the methodological framework is shown in Figure 2.

### 3.1 Prototype decision-making structure

The Delphi technique was proposed by the RAND Corporation (56). Also known as the expert opinion method, it uses back-to-back communication to solicit the predictive opinions of members of an expert group. It is a management technology used to deal with problems involving complex tasks. We use the consensus deviation index (CDI), namely, the mean divided by the variance, to determine consensus in this study.

Step One. Each expert was invited to fill in an open questionnaire according to the prototype decision-making structure above. The experts judged whether the criteria in the decision-making structure were suitable for medical waste logistics, and checked whether the definitions of the criteria were clear based on their knowledge and experience.

Step Two. The experts rated the criteria on a scale of 0–10. The scale indicates the degree of necessity, where a score of “0” indicates that the corresponding factor is absolutely unnecessary and a score of “10” indicates that it is absolutely necessary. We used the CDI to calculate the degree of consensus of the expert group (57).

Step Three. The average values and standard deviations of the scores assigned by all experts to each criterion were calculated. Experts who assigned scores higher than the average ± one standard deviation were asked to explain their reasoning for their scoring before they were allowed to assign new scores.

**Figure 3:**
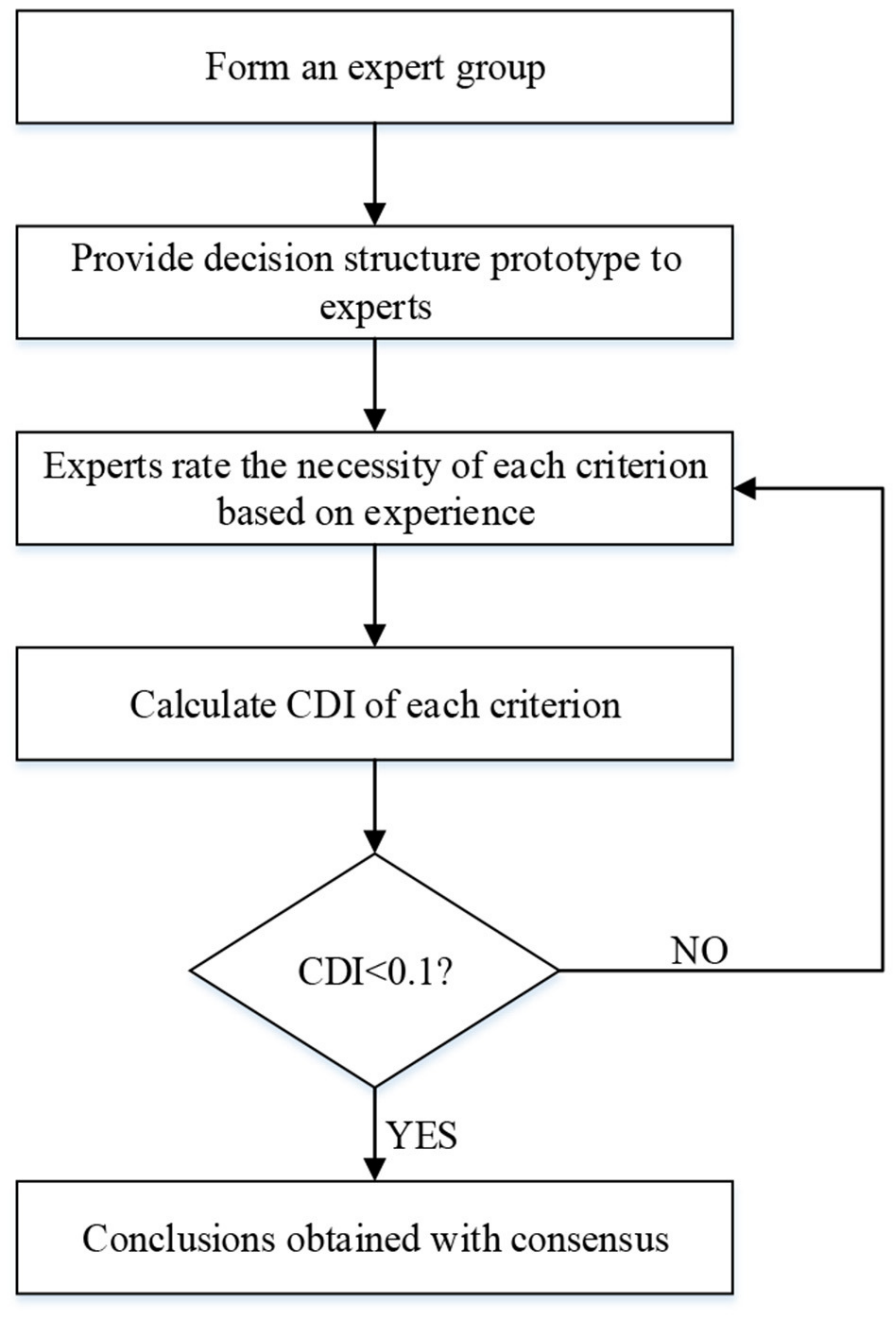
Procedure of the Delphi technique.

If the experts disagreed with one another after the second step, a third step was conducted, and so on until they had reached a consensus. The threshold of the CDI is 0.1. A value higher than 0.1 indicates significant differences in the opinions of experts, and means that further steps of scoring are needed until all the CDI values are lower than 0.1 (58). The procedures involved in the Delphi method are shown in Figure 3.

### 3.2 Best-worst method

The best-worst method (BWM) is an MCDM method developed by Rezaei (59). It can obtain consistent results by using a small amount of information. The BWM has been used to solve many MCDM problems in business, economy, engineering, education, and agriculture, and can be used to determine the weights of factors and select alternatives. Its main steps are as follows:

Step 1: Determine a set of decision criteria.

The decision-maker considers the criteria {*c*_1_, *c*_2_, *c*_3_...*c*_*n*_} that should be used to arrive at a decision.

Step 2: Determine the best (e.g., the most important) and the worst (e.g., the least important) criteria.

The decision-maker identifies the best and the worst criteria. No comparisons are made at this stage.

Step 3: Determine the preference of the best criterion over all the other criteria, and represent it by using a number from 1 to 9. The resulting best-to-others (BO) vector is:


$$\begin{array}{l} A_{B}=\left(a_{B1},a_{B2},a_{B3}\cdots a_{Bn}\right) \end{array}$$

where *a*_*Bj*_ indicates the preference of the best criterion B over criterion *j*. It is clear that *a*_*BB*_ = 1.

Step 4: Determine the preference of all the criteria over the worst criterion and represent it by using a number from 1 to 9. The resulting others-to-worst (OW) vector is:


$$\begin{array}{l} A_{W}={\left(a_{1W},a_{2W},a_{3W}\cdots a_{nW}\right)}^{T} \end{array}$$

where *a*_*jW*_ indicates the preference of criterion j over the worst criterion W. It is clear that *a*_*WW*_ = 1.

Step 5: Calculate the optimal relative weights $\left(w_{1}^{*},w_{2}^{*},\cdots \;,w_{n}^{*}\right)$.

The aim is to determine the optimal weights of the criteria such that the maximum absolute differences $\left|\frac{w_{B}}{w_{j}}-a_{Bj}\right|$ and $\left|\frac{w_{j}}{w_{W}}-a_{jW}\right|$ for all j are minimized. This can translate into the following min–max model:


(1) $$\begin{array}{l} min\;max_{j}\left\{\left|\frac{w_{B}}{w_{j}}-a_{Bj}\right|,\left|\frac{w_{j}}{w_{W}}-a_{jW}\right|\right\}\\ \;\sum_{j}^{s.t.}w_{j}\;=\;1\\ \;w_{j}\;\geq \;0,\;for\;all\;j \end{array}$$

According to literature (60), we minimize the maximum value in the set {|*w*_*B*_ − *a*_*Bj*_*w*_*j*_|, |*w*_*j*_ − *a*_*jw*_*w*_*w*_|}, instead of minimizing the maximum value in $\left\{\left|\frac{w_{B}}{w_{j}}-a_{Bj}|,|\frac{w_{j}}{w_{W}}-a_{jW}\right|\right\}$.

Model (1) can be transferred into the following linear programming model (2) :


(2) $$\begin{array}{l} \;\min\;\zeta^{L}\\ \;s.t.\\ \left|w_{B}\;-\;a_{Bj}w_{j}\right|\;\leq \;\zeta^{L},\;for\;all\;j\\ \left|w_{j}\;-\;a_{jw}w_{w}\right|\;\leq \;\zeta^{L},\;for\;all\;j\\ \;\sum_{j}w_{j}\;=\;1\\ \;w_{j}\;\geq \;0,\;for\;all\;j \end{array}$$

In this model, ξ^*L*^ can be directly considered to be indicator of the consistency of comparisons (we do not use the consistency index here). Values of ξ^*L*^ close to zero reflect a high level of consistency.

Step 6: Find the composite weights $\left(W_{1}^{*},W_{2}^{*},\cdots \;,W_{n}^{*}\right)$.

Once the relative weights of the indicators have been determined, the calculation of the composite weight of the corresponding criterion is simple:


$$\begin{array}{l} W_{i}^{*}=N_{j}*w_{i}^{*}\left(i=1,2,3\ldots n;j=1,2,3\ldots m;n>m\right) \end{array}$$

*W*_*i*_ refers to the composite weight of criterion *i* and *w*_*i*_ is its relative weight. *N*_*j*_ refers to the weight of criterion *i* relative to the corresponding aspect *j*.

### 3.3 Fuzzy evaluation

The fuzzy evaluation method is a comprehensive method of assessment based on fuzzy mathematics (61). It transforms qualitative evaluation into quantitative evaluation based on the membership theory in fuzzy mathematics. It provides clear results, is highly systematic, can be used to solve fuzzy and difficult-to-quantify problems (62), and is thus suitable for a variety of problems involving uncertainty. We use fuzzy vagueness here. The procedure of this method is as follows:

Step 1: Determine the set of factors and their weights.

We construct a set of risk-related factors and calculate their weights. The set can be expressed as:


$$\begin{array}{l} U=u_{1},u_{2},\cdots u_{n} \end{array}$$

Step 2: Determine the set of comments.

We use the literature on the transportation and storage of medical waste to classify the risks posed by it. The comment set *V* is thus formed:


$$\begin{array}{l} V=v_{1},v_{2},\cdots v_{m} \end{array}$$

Step 3: Construct a matrix of comprehensive evaluation.

We used questionnaires for each risk-related factor according to the comment set, collected the responses, and processed them to form a matrix of comprehensive evaluation *R*:


$$R=\left[\begin{array}{cccc} r_{11} & r_{12} & \cdots & r_{1m}\\ r_{21} & r_{22} & \cdots & r_{2m}\\ \vdots & \vdots & \ddots & \vdots \\ r_{n1} & r_{n2} & \cdots & r_{nm} \end{array}\right]$$

where *r*_*nm*_ represents the result of evaluation of factor *n* based on comment *m*.

Step 4: Perform matrix synthesis to obtain the comprehensive set of fuzzy evaluations.

The weighted average operator is a method for performing the synthesis operation. Qian and Xueping (63) used it on seven commonly used synthesis operators (63). We combine the matrix of weights *Z* and the matrix of comprehensive evaluation *R* according to the weighted average operator to obtain the comprehensive set of fuzzy evaluations *B*:


$$B=Z{}^{\circ}R=\left(b_{1},b_{2},\cdots b_{n}\right)\text{=}\sum \left(z_{i}.r_{ij}\right)\left(j=1,2,\ldots m\right)$$

Step 5: Normalize the comprehensive set of fuzzy evaluations.

*B* is normalized. If *B* = (*b*_1_, *b*_2_, ⋯*b*_*n*_), then


$$b_{k}^{\prime }=\frac{b_{k}}{\sum_{j=1}^{m}b_{j}},\left(\forall k\leq m\right)$$

$$B\prime =\left(b_{1}^{\prime },b_{2}^{\prime },\cdots b_{n}^{\prime }\right)$$

Step 6: Perform comprehensive evaluation.

We use the principle of the maximum degree of membership, and choose the corresponding grade *v*_*j*_ of the largest $b_{j}^{\prime }$ in the fuzzy set of comprehensive evaluation $B\prime =\left(b_{1}^{\prime },b_{2}^{\prime },\cdots b_{n}^{\prime }\right)$ as the result.

## 4 Empirical study

### 4.1 Problem statement

Global public health emergencies have begun emerging more frequently in recent years (64). As major cities in China and hubs of international commerce, Beijing, Shanghai, Guangzhou, and Shenzhen have large populations and rich medical resources at all levels, and thus generate large amounts of daily medical waste that requires quick and efficient treatment (65). However, the current number of medical waste disposal enterprises in first-tier cities is small, and all aspects of reverse logistics are not optimal. The management and disposal of MWL in the face of public health emergencies is complex, and needs to be comprehensively improved. We used Beijing, Shanghai, Guangzhou, and Shenzhen as examples in this paper, and examined their capacities for the reverse logistics of medical waste in terms of risk identification, evaluation, and management in case of public health emergencies. The aim is to help government departments in making the correct decisions, protecting people's lives and health, and thus ensuring the healthy development of the urban economy.

### 4.2 Determining the formal decision structure

As is shown in Table 2, we used five experts with rich experience as well as a strong theoretical background in medical waste management, reverse logistics, and emergency management for this case study.

**Table 2:** Professional backgrounds of the selected five experts.

**Expert**	**Organization**	**Position**	**Duties**	**Seniority (yr)**
I	An emergency management bureau	Deputy director of supervision a division	Construct the plan, system, mechanism, and legal framework of emergency management	20
II	A center for disease control and prevention	Deputy director	Emergency response to public health emergencies	18
III	Infection management department of a hospital	Senior technologist	Medical waste management	15
IV	A medical waste recovery and treatment (limited) company	General manager	Recovery and disposal of medical waste	16
V	A centralized hazardous waste disposal center	Medical waste disposal worker	Front-line work of medical waste disposal	8

The preliminary decision structure obtained from the literature review (Table 1) was distributed to the expert group in the first step and their opinions were sought. They were asked to opine whether the classification of each criterion was appropriate and its definition clear.

The experts were asked to rate the necessity of each criterion by assigning it a score from 1 to 10 in the second step. The higher the score of a criterion was, the greater was its necessity/importance. The mean value, variance, and CDI of each criterion were calculated, and the results are shown in Table 3. The CDI values of some standards was >0.1, which means that the expert group had not reached a consensus regarding them and a third step was required.

**Table 3:** Necessity scores of criteria in the second step of the Delphi questionnaire.

**Aspect**	**Criteria**	**Necessity scoring**					**Mean value**	**Standard deviation**	**CDI**
		**I**	**II**	**III**	**IV**	**V**			
Logistics	Transportation route selection	10	9	8	10	8	9	0.894	0.099
	Location of storage site	10	9	9	9	9	9.2	0.400	0.043
	Controllability of in-transit time	8	9	8	8	8	8.2	0.400	0.049
	Entity collaboration	9	9	7	8	7	8	0.894	0.112
	Information transmission channel	8	7	7	8	7	7.4	0.490	0.066
	Conversion and connection of business links	9	9	10	9	9	9.2	0.400	0.043
Emergency capability reserve	Transit capacity reserve	8	10	9	8	9	8.8	0.748	0.085
	Processing capacity reserve	7	8	8	9	7	7.8	0.748	0.096
	Collection and classification capacity reserve	5	6	6	6	7	6	0.632	0.105
	Storage capacity reserve	7	7	8	7	8	7.4	0.490	0.066
Equipment	Packaging safety	8	7	6	7	8	7.2	0.748	0.104
	Transport safety	5	7	7	7	7	6.6	0.800	0.121
	Storage site security	7	8	8	7	8	7.6	0.490	0.064
	Safety of processing equipment	7	8	5	7	8	7	1.095	0.156
	Progressiveness tools	5	6	6	5	5	5.4	0.490	0.091
Personnel risk	Mastery of professional skills	9	8	8	9	8	8.4	0.490	0.058
	Personnel protection awareness	7	8	8	6	7	7.2	0.748	0.104
	Popularity of laws and regulations	7	7	7	7	8	7.2	0.400	0.056
	Professional ethics	6	7	6	6	7	6.4	0.490	0.077
Management risk	Special management laws and regulations	9	9	8	7	8	8.2	0.748	0.091
	Regulatory mechanism	7	6	9	7	6	7	1.095	0.156
	Emergency management measures	10	10	8	10	9	9.4	0.800	0.085
	Process standardization	9	9	9	8	9	8.8	0.400	0.045

The mean value, variance, and CDI of each criterion calculated in the second step were fed back in the third step to the experts, who were asked to explain their scoring of items that exhibited large deviations. After the third step of scoring, the CDI value of each criterion was lower than 0.1, which means that the expert group had reached an agreement regarding all items. In further discussion, the expert group claimed some indicators had a relatively limited impact on the object of research, and thus that indicators with average values smaller than 6 should be discarded. The formal decision-making structure is shown in Table 4.

**Table 4:** Necessity scores of criteria in the third step of scoring of the Delphi questionnaire.

**Aspect**	**Criteria**	**Necessity scoring**					**Mean value**	**Standard deviation**	**CDI**	**Variable number**
		**I**	**II**	**III**	**IV**	**V**				
Logistics (A)	Transportation route selection	10	9	9	10	8	9.2	0.748	0.081	A1
	Location of storage site	10	9	10	9	9	9.4	0.490	0.052	A2
	Controllability of in-transit time	8	9	8	8	8	8.2	0.400	0.049	A3
	Entity collaboration	9	7	9	8	7	8	0.894	0.112	Discarded
	Information transmission channel	8	7	7	8	7	7.4	0.490	0.066	A4
	Conversion and connection of business links	9	9	10	9	9	9.2	0.400	0.043	A5
Emergency capability reserve (B)	Transit capacity reserve	8	10	9	9	9	9	0.632	0.070	B1
	Processing capacity reserve	8	8	8	9	7	8	0.632	0.079	B2
	Collection and classification capacity reserve	5	6	6	6	7	6	0.632	0.105	Discarded
	Storage capacity reserve	7	8	8	7	8	7.6	0.490	0.064	B3
Equipment (C)	Packaging safety	8	7	8	7	8	7.6	0.490	0.064	C1
	Transport safety	6	7	7	7	7	6.8	0.400	0.059	C2
	Storage site security	7	8	8	7	7	7.4	0.490	0.066	C3
	Safety of processing equipment	6	8	5	7	8	6.8	1.166	0.171	Discarded
	Progressiveness tools	5	6	5	5	5	5.2	0.400	0.077	Discarded
Personnel risk (D)	Mastery of professional skills	9	8	8	9	8	8.4	0.490	0.058	D1
	Personnel protection awareness	7	8	8	6	8	7.4	0.800	0.108	Discarded
	Popularity of laws and regulations	7	7	7	7	8	7.2	0.400	0.056	D2
	Professional ethics	7	7	6	6	7	6.6	0.490	0.074	D3
Management risk (E)	Special management laws and regulations	9	9	8	7	8	8.2	0.748	0.091	E1
	Regulatory mechanism	7	6	9	7	6	7	1.095	0.156	Discarded
	Emergency management measures	10	9	8	10	9	9.2	0.748	0.081	E2
	Process standardization	9	9	9	8	9	8.8	0.400	0.045	E3

### 4.3 Identifying key risk-related factors

To identify the key factors influencing MWL and determine the importance of each, we asked the five experts to calculate the absolute weight of each criterion according to the BWM methodology. As an example, we give the procedure used by expert I to perform the above task.

First, expert I selected the most and least important of five aspects—logistics (A), emergency reserve capacity (B) , equipment (C) , personnel-related risk, (D) and management-related risk (E), and assigned scores to them on a scale of 1–9. The results are shown in Table 5.

**Table 5:** Best-to-others (BO) and others-to-worst (OW) pairwise comparison vectors.

BO	A	B	C	D	E
Best criterion: A	1	2	4	8	3
OW	Worst criterion: D				
A	8				
B	6				
C	4				
D	1				
E	6				

Second, expert I selected the most and least important indicators for each of the above five factors. They chose five influential factors, A1, A2, A3, A4, and A5, at the second level for logistics (A), three factors, B1, B2 and B3, for the emergency reserve capacity (B), three influential factors, C1, C2, and C3, for equipment (C), three factors, D1, D2, and D3, for personnel-related risk (D), and three influential factors, E1, E2, and E3, for management-related risk (E), and scored them on a scale of 1–9. The results are shown in Tables 6–10.

**Table 6:** Best-to-others (BO) and others-to-worst (OW) pairwise comparison vectors: aspect A.

BO	A1	A2	A3	A4	A5
Best criterion: A5	8	2	3	6	1
OW	Worst criterion: A1				
A1	1				
A2	6				
A3	4				
A4	2				
A5	8				

**Table 7:** Best-to-others (BO) and others-to-worst (OW) pairwise comparison vectors: aspect B.

BO	B1	B2	B3
Best criterion: B1	1	5	3
OW	Worst criterion: B2		
B1	5		
B2	1		
B3	2		

**Table 8:** Best-to-others (BO) and others-to-worst (OW) pairwise comparison vectors: aspect C.

BO	C1	C2	C3
Best criterion: C1	1	6	4
OW	Worst criterion: C2		
C1	6		
C2	1		
C3	2		

**Table 9:** Best-to-others (BO) and others-to-worst (OW) pairwise comparison vectors: aspect D.

BO	D1	D2	D3
Best criterion: D1	1	5	7
OW	Worst criterion: D3		
D1	7		
D2	2		
D3	1		

**Table 10:** Best-to-others (BO) and others-to-worst (OW) pairwise comparison vectors: aspect E.

BO	E1	E2	E3
Best criterion: E2	8	1	4
OW	Worst criterion: E1		
E1	1		
E2	8		
E3	3		

Finally, the expert used a programming solution in Excel to calculate the relative weights and target values of the above. The closer the target value was to zero, the higher was the consistency among the factors. Their absolute weight is shown in Table 11.

**Table 11:** Weight calculation result of expert I.

**Aspect (weight)**	**ξ^*L*^**	**Criterion**	**ξ^*L*^**	**Relative weight**	**Composite weight**
A (0.4253)	0.0633	A1	0.0500	0.0500	0.0213
		A2		0.2500	0.1063
		A3		0.1667	0.0709
		A4		0.0833	0.0354
		A5		0.4500	0.1914
B (0.2443)		B1	0.0250	0.6500	0.1588
		B2		0.1250	0.0305
		B3		0.2250	0.0550
C (0.1222)		C1	0.0370	0.7037	0.0860
		C2		0.1111	0.0136
		C3		0.1852	0.0226
D (0.0452)		D1	0.0429	0.7429	0.0336
		D2		0.1571	0.0071
		D3		0.1000	0.0045
E (0.1629)		E1	0.0556	0.0833	0.0136
		E2		0.7222	0.1176
		E3		0.1944	0.0317

The results of scores assigned by experts II, III, IV, and V were collected and calculated by using the same steps as above (see Appendix A), and the final weights were obtained by using the geometric mean as shown in Table 12.

**Table 12:** Weight and rank of criterion.

**Criterion**	**Expert I**	**Expert II**	**Expert III**	**Expert IV**	**Expert V**	**Geometric mean**	**Rank**
A1	0.0213	0.0274	0.0209	0.0245	0.0213	0.0229	12
A2	0.1063	0.1095	0.1046	0.1225	0.1914	0.1234	2
A3	0.0709	0.0730	0.0697	0.0817	0.0709	0.0731	6
A4	0.0354	0.0243	0.0418	0.0408	0.0354	0.0350	9
A5	0.1914	0.1825	0.1883	0.2206	0.1063	0.1728	1
B1	0.1588	0.1083	0.0550	0.1380	0.1629	0.1163	3
B2	0.0305	0.0208	0.0305	0.0218	0.0271	0.0258	10
B3	0.0550	0.0375	0.1588	0.0363	0.0543	0.0578	7
C1	0.0860	0.0896	0.0876	0.0863	0.0860	0.0871	5
C2	0.0136	0.0125	0.0122	0.0157	0.0226	0.0149	15
C3	0.0226	0.0229	0.0224	0.0157	0.0136	0.0190	13
D1	0.0336	0.0310	0.0075	0.0364	0.0336	0.0249	11
D2	0.0071	0.0065	0.0339	0.0077	0.0045	0.0089	16
D3	0.0045	0.0042	0.0038	0.0049	0.0071	0.0048	17
E1	0.0136	0.0227	0.0136	0.0123	0.0148	0.0150	14
E2	0.1176	0.1780	0.1176	0.0286	0.1160	0.0960	4
E3	0.0317	0.0492	0.0317	0.1062	0.0321	0.0442	8

Table 12 lists the weights of the criteria according to the index to assess medical waste logistics. For example, the weight of A1 was 0.0229. We ranked all the criteria in descending order of weight to obtain their importance. The results showed that the first eight items were critical factors: A5, A2, B1, E2, C1, A3, B3, and E3.

### 4.4 Assessing the risk to cities

The level of risk of reverse logistics is generally divided into four levels in the literature: significant risk, major risk, general risk, and low risk (59). We distributed 200 questionnaires to stakeholders in MWL in Beijing, Shanghai, Guangzhou, and Shenzhen to investigate this issue further. We collected 185 valid responses from Beijing, 190 from Shanghai, 188 from Guangzhou, and 192 from Shenzhen. We divided the total number of distributed questionnaires by the number of valid questionnaires for statistical processing. For example, Table 13 shows that of the 185 valid respondents from Beijing, 34 claimed that the choice of the transportation route (A1) posed a significant risk to MWL, 56 claimed that it posed a major risk, 78 chose it as a general risk, and 17 determined that it incurred only a low risk. We then divided 34, 56, 78, and 17 by 185 to obtain 0.1838, 0.3027, 0.4216, and 0.0919. This questionnaire was thus convenient and practical, and could adequately reflect the opinions of the respondents. We performed a weighted summation of the results of all questionnaires from all four cities to obtain the final results, and then normalized them as shown in Tables 14–16.

**Table 13:** Scoring results of Beijing.

**Criterion**	**Weight**	**Significant risk**	**Major risk**	**General risk**	**Low risk**
A1	0.0229	0.1838	0.3027	0.4216	0.0919
A2	0.1234	0.2000	0.4108	0.3027	0.0865
A3	0.0731	0.1297	0.1838	0.2432	0.4432
A4	0.0350	0.2432	0.2432	0.1838	0.3297
A5	0.1728	0.1730	0.3514	0.3027	0.1730
B1	0.1163	0.0595	0.3622	0.3027	0.2757
B2	0.0258	0.1243	0.2865	0.2973	0.2919
B3	0.0578	0.2432	0.3622	0.3027	0.0919
C1	0.0871	0.3459	0.4108	0.1730	0.0703
C2	0.0149	0.1730	0.4000	0.3027	0.1243
C3	0.0190	0.0811	0.2432	0.4054	0.2703
D1	0.0249	0.2865	0.2432	0.1730	0.2973
D2	0.0089	0.1730	0.3730	0.1838	0.2703
D3	0.0048	0.1514	0.3622	0.4216	0.0649
E1	0.0150	0.1676	0.2432	0.3027	0.2865
E2	0.0960	0.0973	0.2324	0.2432	0.4270
E3	0.0442	0.1081	0.2324	0.4216	0.2378
Weighted summation		0.1612	0.2705	0.2674	0.2427
Normalization		0.1712	0.3232	0.2821	0.2235

**Table 14:** Scoring results of Shanghai.

**Criterion**	**Weight**	**Significant risk**	**Major risk**	**General risk**	**Low risk**
A1	0.0229	0.1789	0.2895	0.3526	0.1789
A2	0.1234	0.1842	0.3421	0.2368	0.2368
A3	0.0731	0.1211	0.1789	0.4105	0.2895
A4	0.0350	0.1789	0.2368	0.1789	0.4053
A5	0.1728	0.1684	0.1842	0.3368	0.3105
B1	0.1163	0.1211	0.3526	0.2947	0.2316
B2	0.0258	0.1211	0.2789	0.2895	0.3105
B3	0.0578	0.2368	0.3526	0.2947	0.1158
C1	0.0871	0.2895	0.4000	0.1684	0.1421
C2	0.0149	0.1684	0.2421	0.4684	0.1211
C3	0.0190	0.0789	0.2947	0.1789	0.4474
D1	0.0249	0.2789	0.2368	0.1684	0.3158
D2	0.0089	0.1684	0.1684	0.4579	0.2053
D3	0.0048	0.1474	0.3526	0.4105	0.0895
E1	0.0150	0.1632	0.2368	0.2947	0.3053
E2	0.0960	0.0947	0.2263	0.3526	0.3263
E3	0.0442	0.1684	0.2263	0.4105	0.1947
Weighted summation		0.1593	0.2590	0.2823	0.2413
Normalization		0.1691	0.2750	0.2997	0.2562

**Table 15:** Scoring results of Guangzhou.

**Criterion**	**Weight**	**Significant risk**	**Major risk**	**General risk**	**Low risk**
A1	0.0229	0.2287	0.2394	0.2979	0.2340
A2	0.1234	0.1862	0.2287	0.3457	0.2394
A3	0.0731	0.2287	0.2500	0.3936	0.1277
A4	0.0350	0.1915	0.2766	0.3404	0.1915
A5	0.1728	0.2021	0.2819	0.3883	0.1277
B1	0.1163	0.2447	0.1330	0.3085	0.3138
B2	0.0258	0.2500	0.1383	0.3298	0.2819
B3	0.0578	0.2766	0.1436	0.3830	0.1968
C1	0.0871	0.2287	0.1809	0.4309	0.1596
C2	0.0149	0.2979	0.2819	0.2287	0.1915
C3	0.0190	0.1915	0.2394	0.4043	0.1649
D1	0.0249	0.2394	0.2500	0.3617	0.1489
D2	0.0089	0.2500	0.2021	0.3883	0.1596
D3	0.0048	0.2819	0.2447	0.2819	0.1915
E1	0.0150	0.2766	0.2021	0.2500	0.2713
E2	0.0960	0.1915	0.1543	0.2553	0.3989
E3	0.0442	0.1649	0.1596	0.3404	0.3351
Weighted summation		0.2048	0.1963	0.3295	0.2113
Normalization		0.2174	0.2084	0.3499	0.2243

**Table 16:** Scoring results of Shenzhen.

**Criterion**	**Weight**	**Significant risk**	**Major risk**	**General risk**	**Low risk**
A1	0.0229	0.2240	0.1771	0.3542	0.2448
A2	0.1234	0.2344	0.1875	0.3385	0.2396
A3	0.0731	0.2240	0.2292	0.2813	0.2656
A4	0.0350	0.1771	0.2135	0.3854	0.2240
A5	0.1728	0.1406	0.2083	0.4271	0.2240
B1	0.1163	0.2344	0.1979	0.3958	0.1719
B2	0.0258	0.3385	0.2813	0.2396	0.1406
B3	0.0578	0.2344	0.2240	0.3906	0.1510
C1	0.0871	0.2865	0.1198	0.4010	0.1927
C2	0.0149	0.3177	0.2813	0.3333	0.0677
C3	0.0190	0.1458	0.2240	0.3385	0.2917
D1	0.0249	0.1563	0.3333	0.3906	0.1198
D2	0.0089	0.2240	0.2917	0.3385	0.1458
D3	0.0048	0.2448	0.3854	0.2917	0.0781
E1	0.0150	0.2656	0.3385	0.2344	0.1615
E2	0.0960	0.1823	0.2344	0.3958	0.1875
E3	0.0442	0.2188	0.2813	0.2344	0.2656
Weighted summation		0.2011	0.2023	0.3449	0.1936
Normalization		0.2135	0.2148	0.3661	0.2056

The results in the above tables show that MWL posed a major risk in Beijing and a general risk in Shanghai, Guangzhou, and Shenzhen. Both general and low levels of risks are generally considered acceptable while significant and major levels of risk are deemed unacceptable.

## 5 Discussion and conclusions

### 5.1 Discussion

We identified the risks of MWL in case of public health emergencies and ranked the risk-related factors according to their importance in this study. The results showed that linking business processes is the most critical risk in the above context. Previous studies have claimed that the most critical risk posed by MWL lies in transportation and storage (5). The results of this study are different from this. According to historical data on accidents involving medical waste in Beijing in recent years, 40% of them occurred during business handover while only < 10% occurred during transportation and storage. The results of our study differ from those of past work because the latter was based on older data. The technological capabilities and skills of management of enterprises involved in MWL have improved with awareness of it. However, the participants of MWL include hospitals, transportation companies, disposal institutions, and other organizations. Some of them are public institutions and others are profit-making enterprises. Their operational objectives are thus different such that they are usually responsible only for achieving their own goals, and have little incentive to cooperate with one another. The handing and taking over of activities in the process may also involve personnel who are not adept at the task.

The location of storage sites is related to efficiency and security in the MWL network. While many studies have investigated site selection (45), a number of factors need to be considered in this vein. When the constraints of time, cost, and service satisfaction cannot be simultaneously met, the importance-based ranking of the risk-related factors can help arrive at a satisfactory solution. The capacity for emergency transportation is also an important risk-related factor. Zhihao et al. (66) recognized its important role (66) but did not consider it to be a key risk. Our survey here showed that the frequency of transportation increases significantly in case of public health emergencies, and the path of transportation of waste changes often based on the available equipment, temporary storage sites, and disposal locations. Flexibility in the capacity for emergency transportation is thus necessary. It plays a critical in the stability of MWL as a bridge among collection, storage, and disposal.

Due to advances in packaging technology, some researchers have claimed that the safety of packaging equipment is no longer a key risk (6). We disagree. There is great deal of uncertainty in the type and quantity of medical waste that is generated when public health emergencies arise. Different types of medical waste require different packaging, and this may lead to a mismatch between the available packaging and the medical waste that needs to be sealed. In addition, the packaging operation usually occurs at the source of waste discharge, but the workers at these facilities are usually not skilled in packaging operations. This is one reason for why packaging remains a risk in MWL.

Our work here has shown that management-related and personnel-related risks are not key in MWL. While management and personnel training are important for daily MWL, their roles are not significant when public health emergencies are considered. This is because establishing the standards of management and personnel training requires a certain lead time, that is, emergency drills are more important than temporary responses.

### 5.2 Risk control measures

According to the importance of the risk-related factors calculated above, we propose the following measures to control the risk posed by MWL:

(1) The linking of the relevant business processes must be prioritized because the risk posed by it directly affects the basic operation of MWL, and public health emergencies can amplify its impacts. The government should help the participants in the MWL process to cooperate by integrating the supply chain. First, the government should specify the division of responsibilities of each participant to MWL and set the standards of operation for them. Second, it is necessary to help build information-sharing platforms for them. When a public health emergency occurs, all participants of MWL should communicate with one another about the problems encountered, share the risks, and jointly propose solutions.
(2) The risk posed by the location of waste storage sites can be mitigated through coordination between the government and the relevant private enterprises. It is difficult for enterprises to resolve the conflict between cost and efficiency without the support of the government. In addition, the government needs to plan for temporary locations for storage sites in advance, and to conduct regular mock tests of the overall operation to prepare for public health emergencies.
(3) The government needs to plan for its capacity for emergency transportation in advance, and to encourage enterprises to transform non-specialized medical waste equipment and train an appropriate number of personnel to account for emergencies based on subsidies and other incentives. This can help ensure that the requisite resources can be quickly mobilized in case of public health emergencies.
(4) The risk posed by the safety of packaging should also be considered. Transportation enterprises need to acquire the relevant knowledge on medical waste while hospitals need to learn the appropriate packaging and classification of medical waste. In addition, the government needs to support scientific research institutions in developing safer packaging to help hospitals conveniently deal with medical waste.
(5) Personnel need to be trained in emergency preparedness and a plan for emergency management needs to be formulated in advance. A scientific warning mechanism can also buy more preparation time for the relevant departments in case of public health emergencies.

### 5.3 Conclusions

This article considers the impact of public health emergencies on MWL and researches risk identification and control of MWL from the perspective of systemic coordination. This article combines multiple disciplines, such as environmental science, public health, and logistics management, to provide practical cases for interdisciplinary research. By analyzing and controlling the risks in the logistics process of medical waste, the development of risk management theory can be promoted, especially in high-risk and highly sensitive fields. This article helps to improve relevant policies and regulations, providing a scientific basis for policymakers.

We used the BWM to obtain the weights and ranked importance of risk-related factors in MWL and found that linking the relevant business processes, location of storage sites, capacity for emergency transportation, emergency management measures, and safety of packaging are the key risk-related factors in MWL. The used four first-tier cities in China as examples, and assessed the risk posed to them by MWL. Their final ranking in terms of risk was Beijing > Shanghai > Guangzhou > Shenzhen. Such results are useful for the construction of MWL. Effective risk control measures include integrating supply chain management, emergency training for personnel, training in waste packaging, and an early warning mechanism for public health emergencies.

The disposal standards and processes about medical waste vary in different countries, so the risks posed by MWL are also different. The prototype for decision making structure in this paper sorted and classified the risks posed by MWL in public health emergencies, and it was of high universality. By expanding the number of experts to clarify the selection criteria, the formal decision-making structure can be better matched with the regional characteristics of the evaluation object. In addition, this paper took Beijing as an example and proposed risk control measures for key risks. However, in practice, further consideration needs to be given to the implementation costs of the measures, while also taking into account the coordination effect between different measures. In addition, early risk control is also a future research direction. By combining key risk factors with data analysis techniques to design an MWL risk warning model and developing response strategies based on different risk scenarios, the resilience of MWL in major health and safety incidents can be further enhanced.

## Data availability statement

The original contributions presented in the study are included in the article/supplementary material, further inquiries can be directed to the corresponding author.

## Author contributions

XW: Conceptualization, Writing – original draft. LL: Data curation, Writing – review & editing. LW: Data curation, Writing – original draft, Writing – review & editing. WC: Resources, Supervision, Writing – original draft. DG: Methodology, Validation.
