# Supplementary material for: An application of BWM for risk control in reverse logistics of medical waste
Source: Front Public Health. 2024 Jan 26;12:1331679. doi: 10.3389/fpubh.2024.1331679 (PMC10853444; doi:10.3389/fpubh.2024.1331679)
Supplement: Supplementary file 1 [file Data_Sheet_1.docx]

# Appendix A

Table A1 Weight calculation result of expert Ⅱ

| Aspect(weight) | $\xi^{L}$ | Criterion | $\xi^{L}$ | relative weight | composite weight |
| --- | --- | --- | --- | --- | --- |
| A(0.4167) | 0.0833 | A1 | 0.0876 | 0.0657 | 0.0274 |
|  |  | A2 |  | 0.2628 | 0.1095 |
|  |  | A3 |  | 0.1752 | 0.0730 |
|  |  | A4 |  | 0.0584 | 0.0243 |
|  |  | A5 |  | 0.4380 | 0.1825 |
| B(0.1667) |  | B1 | 0.0250 | 0.6500 | 0.1083 |
|  |  | B2 |  | 0.1250 | 0.0208 |
|  |  | B3 |  | 0.2250 | 0.0375 |
| C(0.1250) |  | C1 | 0.0167 | 0.7167 | 0.0896 |
|  |  | C2 |  | 0.1000 | 0.0125 |
|  |  | C3 |  | 0.1833 | 0.0229 |
| D(0.0417) |  | D1 | 0.0429 | 0.7429 | 0.0310 |
|  |  | D2 |  | 0.1571 | 0.0065 |
|  |  | D3 |  | 0.1000 | 0.0042 |
| E(0.2500) |  | E1 | 0.0758 | 0.0909 | 0.0227 |
|  |  | E2 |  | 0.7121 | 0.1780 |
|  |  | E3 |  | 0.1970 | 0.0492 |

Table A2 Weight calculation result of expert Ⅲ

| Aspect(weight) | $\xi^{L}$ | Criterion | $\xi^{L}$ | relative weight | composite weight |
| --- | --- | --- | --- | --- | --- |
| A(0.4253) | 0.0633 | A1 | 0.0492 | 0.0492 | 0.0209 |
|  |  | A2 |  | 0.2459 | 0.1046 |
|  |  | A3 |  | 0.1639 | 0.0697 |
|  |  | A4 |  | 0.0984 | 0.0418 |
|  |  | A5 |  | 0.4426 | 0.1883 |
| B(0.2433) |  | B1 | 0.0250 | 0.2250 | 0.0550 |
|  |  | B2 |  | 0.1250 | 0.0305 |
|  |  | B3 |  | 0.6500 | 0.1588 |
| C(0.1222) |  | C1 | 0.0167 | 0.7167 | 0.0876 |
|  |  | C2 |  | 0.1000 | 0.0122 |
|  |  | C3 |  | 0.1833 | 0.0224 |
| D(0.0452) |  | D1 | 0.0833 | 0.1667 | 0.0075 |
|  |  | D2 |  | 0.7500 | 0.0339 |
|  |  | D3 |  | 0.0833 | 0.0038 |
| E(0.1629) |  | E1 | 0.0556 | 0.0833 | 0.0136 |
|  |  | E2 |  | 0.7222 | 0.1176 |
|  |  | E3 |  | 0.1944 | 0.0317 |

Table A3 Weight calculation result of expert Ⅳ

| Aspect(weight) | $\xi^{L}$ | Criterion | $\xi^{L}$ | relative weight | composite weight |
| --- | --- | --- | --- | --- | --- |
| A(0.4902) | 0.0980 | A1 | 0.0500 | 0.0500 | 0.0245 |
|  |  | A2 |  | 0.2500 | 0.1225 |
|  |  | A3 |  | 0.1667 | 0.0817 |
|  |  | A4 |  | 0.0833 | 0.0408 |
|  |  | A5 |  | 0.4500 | 0.2206 |
| B(0.1961) |  | B1 | 0.0370 | 0.7037 | 0.1380 |
|  |  | B2 |  | 0.1111 | 0.0218 |
|  |  | B3 |  | 0.1852 | 0.0363 |
| C(0.1176) |  | C1 | 0.2000 | 0.7333 | 0.0863 |
|  |  | C2 |  | 0.1333 | 0.0157 |
|  |  | C3 |  | 0.1333 | 0.0157 |
| D(0.0490) |  | D1 | 0.0428 | 0.7429 | 0.0364 |
|  |  | D2 |  | 0.1571 | 0.0077 |
|  |  | D3 |  | 0.1000 | 0.0049 |
| E(0.1471) |  | E1 | 0.0556 | 0.0833 | 0.0123 |
|  |  | E2 |  | 0.1944 | 0.0286 |
|  |  | E3 |  | 0.7222 | 0.1062 |

Table A4 Weight calculation result of expert Ⅴ

| Aspect(weight) | $\xi^{L}$ | Criterion | $\xi^{L}$ | relative weight | composite weight |
| --- | --- | --- | --- | --- | --- |
| A(0.4253) | 0.0633 | A1 | 0.0500 | 0.0500 | 0.0213 |
|  |  | A2 |  | 0.4500 | 0.1914 |
|  |  | A3 |  | 0.1667 | 0.0709 |
|  |  | A4 |  | 0.0833 | 0.0354 |
|  |  | A5 |  | 0.2500 | 0.1063 |
| B(0.2443) |  | B1 | 0.0000 | 0.6667 | 0.1629 |
|  |  | B2 |  | 0.1111 | 0.0271 |
|  |  | B3 |  | 0.2222 | 0.0543 |
| C(0.1222) |  | C1 | 0.0370 | 0.7037 | 0.0860 |
|  |  | C2 |  | 0.1852 | 0.0226 |
|  |  | C3 |  | 0.1111 | 0.0136 |
| D(0.0452) |  | D1 | 0.0429 | 0.7429 | 0.0336 |
|  |  | D2 |  | 0.1000 | 0.0045 |
|  |  | D3 |  | 0.1571 | 0.0071 |
| E(0.1629) |  | E1 | 0.0758 | 0.0909 | 0.0148 |
|  |  | E2 |  | 0.7121 | 0.1160 |
|  |  | E3 |  | 0.1970 | 0.0321 |
